# By-Products Revalorization with Non-Thermal Treatments to Enhance Phytochemical Compounds of Fruit and Vegetables Derived Products: A Review

**DOI:** 10.3390/foods11010059

**Published:** 2021-12-27

**Authors:** Marina Cano-Lamadrid, Francisco Artés-Hernández

**Affiliations:** 1 Food Quality and Safety Group, Department of Agrofood Technology, Universidad Miguel Hernández, Ctra. Beniel, Km 3.2, Orihuela, 03312 Alicante, Spain; 2 Postharvest and Refrigeration Group, Department of Agronomical Engineering and Institute of Plant Biotechnology, Universidad Politécnica de Cartagena, Cartagena, 30203 Murcia, Spain; fr.artes-hdez@upct.es

**Keywords:** zero waste, bioactive compounds, green technologies, nutraceuticals, circular economy

## Abstract

The aim of this review is to provide comprehensive information about non-thermal technologies applied in fruit and vegetables (F&V) by-products to enhance their phytochemicals and to obtain pectin. Moreover, the potential use of such compounds for food supplementation will also be of particular interest as a relevant and sustainable strategy to increase functional properties. The thermal instability of bioactive compounds, which induces a reduction of the content, has led to research and development during recent decades of non-thermal innovative technologies to preserve such nutraceuticals. Therefore, ultrasounds, light stresses, enzyme assisted treatment, fermentation, electro-technologies and high pressure, among others, have been developed and improved. Scientific evidence of F&V by-products application in food, pharmacologic and cosmetic products, and packaging materials were also found. Among food applications, it could be mentioned as enriched minimally processed fruits, beverages and purees fortification, healthier and “clean label” bakery and confectionary products, intelligent food packaging, and edible coatings. Future investigations should be focused on the optimization of ‘green’ non-thermal and sustainable-technologies on the F&V by-products’ key compounds for the full-utilization of raw material in the food industry.

## 1. Introduction

The Food and Agriculture Organization (FAO) of the United Nations indicates that around a third of all food production is globally lost or wasted at some point in the food chain [1,2]. Losses vary a lot depending on the chain considered and in the case of fruit and vegetables (F&V) can reach up to 50%. Within the F&V processing operations about 25% to 30% of waste is produced [3]. The most important causes of losses on farms include inappropriate timing for harvesting, overproduction, underutilized products, climatic conditions, harvesting and handling practices, and inadequate postharvest technology [4]. At the World Food Summit held in 2017 organized by FAO, the challenges needed to achieve food stability and food availability were identified and a roadmap was proposed to reduce 50% of food waste by 2050. The principles of eco-innovation are the industrial ecology and the circular economy (“zero waste” and the use of wastes as raw materials) [5]. Among the challenges that arise different actions stand out, such as the revaluation of waste in the various stages of the production process and logistics, and/or the use of waste products (by-products) as starting raw material for the production of products with greater added value [6] and then called co-products.

The handling and processing of these raw materials generates a large number of commodity by-products being undervalued and underused, and although there are some minor uses such as the production of biomass and animal feed, these strategies do not guarantee an efficient use of this material that could offer interesting possibilities for the agri-food industry and the reduction of this environmental problem [2,7]. Horticultural by-products mainly are peels, pomace and seeds, which could be a potential good source of bioactive compounds with high added-value such as pectins, proteins, polysaccharides, flavor compounds, dietary fibers, and phytochemicals compounds [8]. To continue being relevant, it is necessary to further strengthen and dynamize the sector through the development of appropriate postharvest strategies to increase shelf life, and a model for the enhancement of horticultural by-products through the incorporation of emerging and sustainable ‘Green Technologies’ to its revalorization [9]. The strategies to revalue horticultural by-products can lead to a change in the productive model of the sector and evolve towards a more diversified and sustainable circular economy, giving more added value and competitiveness. These strategies can be focused on obtaining potential ingredients for the food industry, cosmetics, and/or the pharmaceutical industry. The use of plant by-products supports the low-carbon economy by using renewable resources, offering environmental and economic benefits and improving efficiency in the food industry [7,10].

Nowadays, the tendency in the food market is driven by different reasons such as health and sustainability. This phenomenon is expressed in the consumer’s interest in healthy natural foods based on plant products. Food producers are increasingly striving to meet these trends by offering “Clean label” foods or ingredients. Currently, there is no legislation related to the aforementioned concept, but the growing demand for this type of food reflects the desire of consumers for food to be more “natural”, wholesome, premium, and use environmentally friendly technologies [11,12]. The extracts obtained from F&V by-products can fulfill a series of technological functions such as being colorants, antioxidants, flavors or antimicrobial agents, or act directly as ingredients to enrich or improve the functional properties of some food becoming a supplemented or fortified commodity [8,13,14,15,16].

In order to obtain value-added compounds with functional (nutraceuticals) and techno-functional (pigments) properties, technologies have been developed for each side-product generated from agro-food industries [17]. Conventional and traditional thermal methods are still in use, although high energy consumption, the degradation of thermolabile nutritional compounds, and sensory quality changes occur, which require the adoption of sustainable preservation techniques without altering the sensory and nutritional quality of foods [18]. The stability of nutraceuticals is affected by different factors (temperature, pH, light stress, presence or absence of oxygen, and enzymatic activity). Focusing on temperature factor, there is increased interest in improving and optimizing non-thermal technologies to avoid degradation of key compounds, jointed with sustainable methods [19]. Among non-thermal technologies, the most common are ultrasound-assisted extraction, high-pressure processing, light stresses, fermentation technology, electro-technologies, and enzyme-assisted extraction. More detailed information is described in Section 3.

Therefore, this review is focused on generating comprehensive information about non-thermal technologies applied in F&V by-products to enhance phytochemical compounds such as polyphenols, pigments and nitrogen/organosulfur derivates, and to obtain pectin. Moreover, the potential use of such compounds will also be of a particular interest to this review.

## 2. Fruit and Vegetables By-Products as a Source of Valuable Compounds

Scientific research and development have been greatly increased in the last decades in the field of extraction and the application of bioactive compounds re-valorized from F&V processing by-products [20]. A large number of molecules with added-value (simple sugars, carbohydrates, polysaccharides, pectin, fibers, phenolic acids, carotenoids, tocopherols, flavonoids, vitamins and aromatic compounds) from F&V by-products can be used in the food, cosmetic, or pharma industry (co-products) [19,21,22]. This review will be focused on phytochemical compounds such as polyphenols, pigments, sulfur compounds, and pectins. Nowadays, manufactures are focused on reducing the environmental impact of industrial by-products (zero waste and circular economy) and recovering bioactive compounds from agricultural by-products. 

### 2.1. Phytochemical Compounds

**Figure 1:**
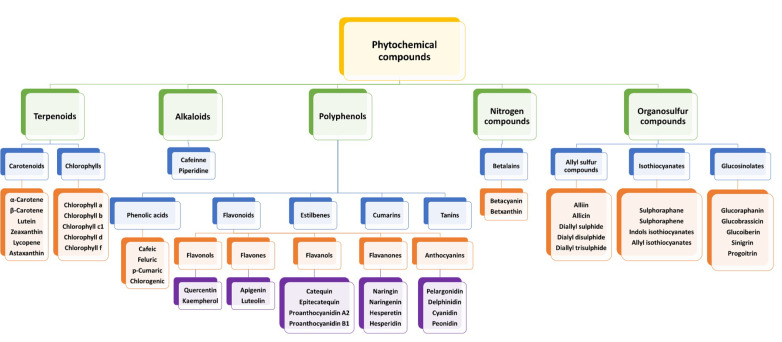
Classification of the main phytochemical compounds in fruit and vegetables (F&V) by-products.

Phytochemicals are defined as compounds obtained from plants, naturally biosynthesized in their secondary metabolism without any essential nutritional values. However, they present lots of health promoting properties according to their biological activity [23]. They are used for several purposes such as drugs, agrochemicals (biopesticides), and food additives (aroma, colorant agents). Phytochemicals were divided into different groups such as terpenoids (carotenoids and chlorophylls), polyphenols, alkaloids, nitrogen compounds, and organosulfur compounds (Figure 1). Three main subsections were made focusing on the greatest relevance groups for this review work: polyphenols (Section 2.1.1), pigments (Section 2.1.2) and organosulfur compounds (Section 2.1.3). 

#### 2.1.1. Polyphenols

The most common key compounds from F&V by-products are polyphenols (Figure 1: phenolic acids and their polymeric derivatives, such as lignans, stilbenes, tannins, and flavonoids) in skins, pulp, seeds, or pomace [24]. Phenolic acids are common in F&V by-products such as apple pomace (chlorogenic acid, and cryptochlorogenic acid) [25], artichoke (bracts, leaves and stems) (chlorogenic acid) [26], mango kernel and leaves (gallic acid, and ellagic acid) [27], pomegranate peel (caffeic acid, chlorogenic acid, ellagic acid, and gallic acid) [28], potato peel (chlorogenic acid, ferulic, gallic, protocatechuic and caffeic acid) [29], tomato peel (3-caffeoylquinic acid, 5-caffeoylquinic acid, 3,4-di-O-caffeoylquinic acid, and 3,4,5-tri-caffeoylquinic acid) [30] and blueberry pomace (cinnamic acid derivatives) [22,31,32,33]. Among flavonoids, flavones, flavanones, anthocyanidins, and flavonols can be found in grape pomace (catechins, anthocyanins, stilbenes, and flavonol glycosides), onion skin (quercetin 3,40-O-diglucoside and quercetin 4-o-monoglucoside and isorhamnetin-3-glucoside) [34], tomato peel (lycopene, naringenin chalcone and naringenin) [30], apple pomace (hydroxycinnamates, phloretin glycosides, quercetin glycosides, catechins, procyanidins, and epicatechin) [25], figs peel (cyanidin-3-rutinoside, cyanidin-3,5-diglucoside, cyanidin-3-O-diglucoside, epitecatechin, catechin and quercetin-rutinoside) [32], blueberry pomace (anthocyanins and flavonol-glycosides) [33], and citrus peel (eriocitrin, hesperidin, and naringin) [22,31,32,35]. Some of them are pigments such as anthocyanins, and are explained in Section 2.1.2. jointed with other pigments (betalains, carotenoids, and chlorophylls).

#### 2.1.2. Bioactive Pigments

**Figure 2:**
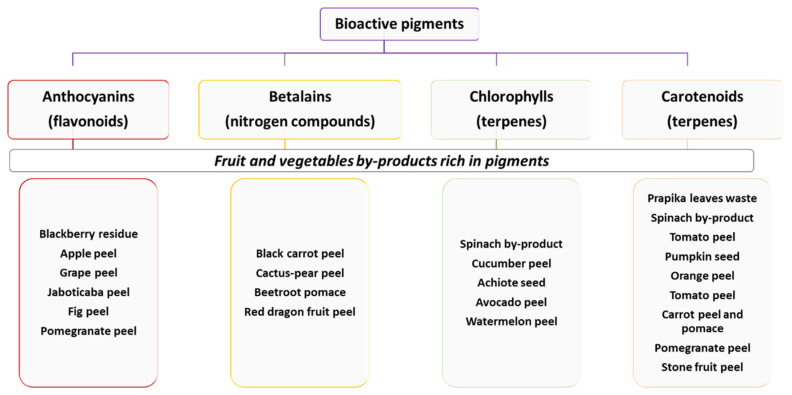
Types of the main bioactive pigments and examples of some fruits and vegetable (F&V) by-products rich in these pigments [8,32,38,39].

Plant pigments are colored substances produced by plants and are important in controlling photosynthesis, growth, and development [36]. The market for natural colorants is experiencing a boom related to the “clean label” trend. It is worth mentioning that some of the main drivers for the increased demand of natural colorants are the health-promoting benefits of natural food colorants [37]. Researchers and the food industry are exploring stable natural colorants and new natural extracts from F&V by-products [37]. These by-products tissues are rich in betalains, anthocyanins, carotenoids, or/and chlorophylls. Figure 2 shows the classification of bioactive pigments and some examples of commodity by-products rich in these pigments.

##### Water-Soluble Compounds: Anthocyanins and Betalains

Flavonoids are a group of secondary metabolites which belong to the class of phenylpropanoid and present a wide color range, from pale-yellow to blue. Among them, anthocyanins are responsible for the orange-to-blue colors; different parts of the plant present these compounds such as leaves, fruits, and seeds, among others. Wineries and juice manufacturer by-products are enriched sources for anthocyanin pigments that can be used as natural colorants for various food applications [38]. The use of anthocyanins as pigments (E-163) is accepted by the European Community [40].

Betalains are yellow-to-red nitrogen-containing compounds, derived from tyrosine. The use of betalains as pigments (E-162) is also accepted by the European Community and they are used in the production of jellies, jams, strawberry yogurt, among other products [41]. Betalains come from the underutilized biomass of red beetroot processing and from beetroot leaves [38,42].

##### Fat-Soluble Compounds: Carotenoids and Chlorophylls

Carotenoids are isoprenoids, and essential compounds of the photosystems in plants. They are responsible for the yellow-red coloration. Up to now, commercially available carotenoids synthesized chemically are being used as coloring compounds [43]. However, currently, these pigments can be obtained from F&V by-products. Carotenoids are often located in the same plant organs as anthocyanins, increasing color variety when they combine [43]. 

On the other hand, although chlorophylls can be used for coloring food products, there are limited available scientific reports on the use of F&V by-products for the extraction of chlorophylls and their further application as a colorant in food formulations [38]. 

#### 2.1.3. Sulfur Compounds 

Sulfur is an essential compound for the biosynthesis of phytoalexins, sulfur-containing glycosides (glucosinolates), and alliins, among others. Alliaceous (onion, garlic) and cruciferous vegetables (broccoli, cauliflower, radish, cabbage) are the main sources of sulfur compounds (contributed up to 42% of total sulphur intake) [44]. These metabolic compounds play a vital role in the physiology and protection of plants against several environmental stresses [45]. The alliaceous and brassica by-products (for example Bimi leaves [13] and broccoli by-products [46]) contain mainly glucosinolates as sulfur compounds. Glucosinolates can be found as not biologically active unhydrolysed compounds. However, these by-products present the myrosinase enzyme which produces several biologically active isothiocyanates and indoles, with health potential properties such as chemopreventive activity against cancer. Among them, sulforaphane is the most researched isothiocyanate from the degradation of glucoraphanin [47]. Bioactive sulfur compounds are degraded during processing, mainly by conventional thermal techniques. Even some of these compounds could not be formed by inactivation of the myrosinase enzyme.

### 2.2. Pectins

Pectin is a structural hetero-polysaccharide contained in the cell walls and abundant in the non-woody parts of plants, including by-products such as peel or pomace. Pectin presents beneficial properties for humans such as moderating the glycemic index and slowing gastric transit. The interaction of pectin and polyphenolic compounds contributes to systemic anti-inflammation [48]. Pectin is widely used in the food industry as a gelling agent, emulsifier, and carrier polymer for the encapsulation of food ingredients (it is an effective delivery vehicle for exogenous nutraceuticals), helping protect and promote the controlled release of biomolecules [48]. Pectin quality can be characterized by galacturonic acid content, degree of esterification and degree of methylation, affecting gelling properties [49]. Recent research summarized the characterization of the pectin composition of several F&V waste, especially form plant processing industry. One of the conclusions was that the pectin structures and recovery vary depending on the source and the applied extraction pectin as it can be observed in the Section 7 (focus on non-thermal technologies). Moreover, the information about changes in pectic polysaccharide composition after processing is essential for the industry, including the amount of uronic acid due to the requirement of the minimum of 65%. In addition, although more studies are needed, the rest of pectin below this limit could be useful in other applications [50].

## 3. Potential and Innovative Non-Thermal Techniques for Revalorization of Fruit & Vegetables By-Products

Due to the thermal instability of compounds (which means a reduction of their concentration level), non-thermal innovative technologies have been increasing during last decades [19], such as ultrasound-assisted extraction, high-pressure processing, light stresses, fermentation technology, electro-technologies, and enzyme-assisted extraction, among others [51,52]. Most of them are focused on the recovery of the above-mentioned compounds related to revalorization of F&V by-products [9,24]. Recovering of bioactive phytochemicals from F&V waste by non-thermal processes could improve the efficient production of potential bioactive ingredients [53].

### 3.1. Electro-Technologies: Pulses Electric Fields

Pulses Electric Fields (PEF) consist of subjecting the selected material to the intermittent application (<300 Hz) of electric fields at moderate-high intensity (0.1–20 kV/cm) and short duration (µs to ms) [54]. The main characteristic is the application of electric field pulsing on plant matrices that induces electro permeabilization (formation of located pores in cell membranes of cells), and the effect mainly depends on medium composition (conductivity) [55]. PEF technology has been defined as technology which requires fewer resources to produce nutritional with optimal sensory characteristics and longer shelf lives of products such as hummus, smoothies and juices [56]. Related to recovery bioactive compounds from F&V by-products, it enhances the specific recovery of bioactive intracellular compounds without increasing temperature or/and damaging the structure of the matrix. The obtained result depends on treatment intensity, physicochemical properties of the matrix and the tissues and cells composition. If the combination of the variables is optimized, reversible electroporation could occur (the membrane can return to its original state once the electric field application has finished) [54,57,58]. It is important to highlight that recent study indicated that pulsed electric field (PEF) treatment needs an optimization for more selective, quicker, and sustainable bio-active compounds extraction in the food industry [58]. Therefore, recent information about the optimal conditions of PEF were included in Tables 1–3.

### 3.2. Enzyme-Assisted Extraction

A novel green and non-thermal technology, enzyme-assisted technology, for bioactive compounds extraction such as phenolics and pectin has been developed during last decades for cosmetic, pharmaceutical and food applications. It is essential to highlight that enzyme-assisted extraction allows the use of F&V by-products providing a novel chance to give added-value to F&V waste [59]. The fundamental mechanism of the pectin, polyphenols, and pigments enzyme-assisted extraction from F&V by-products is based on the cell-wall degrading enzymes. These enzymes weaken, degrade partially or/and break down the cell wall polysaccharides, enhancing the possibility of the extraction of those compounds [60].

### 3.3. Fermentation

Fermentative processes can be classified according to different criteria. One of the most common is the group of batch fermentations which is based on the addition of the substrate and the key microorganism in the system at time zero. The produced key compounds cannot be obtained until the process is complete [61]. On the other hand, continuous and fed-batch fermentations microorganisms present another mechanism. The system can be reutilized for several batches, increasing its efficiency. In general, the industrial fermentations take place in liquid media, but sometimes solid-state fermentations microorganisms are applied. Related to fermentations and revalorizations of F&V by-products (fermentation-based valorization strategies), it has been recently developed the fermentation of date palm waste to produce lactic acid [62,63] and bioconversion of cocoa by-products using different microorganisms to obtain key enzymes, among other bioactive compounds [63,64].

### 3.4. High Hydrostatic Pressure 

High Hydrostatic Pressure (HPP) is one of the non-thermal pasteurization processing technologies which is widely applied in the food industry [51,52]. It is a processing technique that uses a range of pressure from 100 to 900 MPa to increase shelf-life of the products due to the inactivation and elimination of microorganisms. The pressure can be applied through direct pressure and indirect pressure. HPP induces high pressure which causes severe damage to plant cells and leads to the diffusion of solvents and enhances the mass transfer and release of the extracts [65]. The uniformity of the pressure application is maintained during the process and it does not depend on the product size and geometry. It has been reported that this technique avoids no-desirable effects on texture characteristics. Moreover, this technique does not reach high temperatures, then protect characteristic flavor notes, color pigments nutrients, and antioxidant bioactive compounds which are degraded at high temperatures [66].

### 3.5. Light Stress 

Plant by-products have been proposed as bio-factories of bioactive compounds through different induced postharvest abiotic stress mechanisms. Among them, one of the most promising techniques appears to be UV radiation, the spectrum is divided into three regions: UV-A (wavelength 320 to 400 nm), UV-B (wavelength 280 to 320 nm) and UV- C (wavelength 220–280 nm). The use of UV technology during post-harvest is an emerging technology to enhance the biosynthesis of bioactive compounds in the F&V industry, respectful with the environment, without generating waste [67,68]. The application of UV-B, alone or in combination with UV-C, has not been widely studied as a revalorization tool for maintaining and/or increasing the main key compounds in F&V by-products [69]. Although light-emitting diodes (LEDs) are increasingly adopted for the production of several vegetable modalities and for quality preservation during storage [70], influencing the metabolic pathways (biosynthesis of several bioactive compounds) [71,72,73]. No published information is already available concerning the effect of this light stress in F&V by-products. Recently, it has been concluded that a combination of different light stress techniques (UV-B + LEDs) could be a good strategy to enhance the bioactive compounds in commodities, being a potential tool for by-products revalorization [71].

### 3.6. Supercritical Fluid Extraction

Supercritical fluid extraction (SFE) is a recent extraction technique, and it is based on the use of the critical point of the solvent during the extraction. The combination of gas mass transfer and liquid solvation properties allows a high transfer mass (diffusion coefficients) than working below critical point. The majority of SFE studies have focused on the use of CO_2_ due to its characteristics (non-toxic and cheap and can be easily removed after extraction) [74].

### 3.7. Ultrasound-Assisted Extraction (UAE)

Ultrasonication is an emerging non-thermal and green technology in the food sector, although it has been previously established in other sectors such as pharmacological. The fundamentals are based on the mechanical impact of the ultrasound waves, allowing deeper penetration of the solvent into the matrix (“sponge effect”) [55]. Ultrasonication can be used with different doses (frequencies and time), which are classified as: (i) low-frequency (20 kHz–100 kHz); (ii) medium-frequency (100 kHz–1 MHz); and high-frequency ultrasonication (1 MHz–100 MHz) [75,76]. In food processing, the most common frequency range for the extraction of bioactive compounds and intensified synthesis is 20 kHz–100 kHz [51,52,77].

## 4. Scientific Literature Review about Non-Thermal Technologies Used for Revalorization of Fruits & Vegetable By-Products

**Figure 3:**
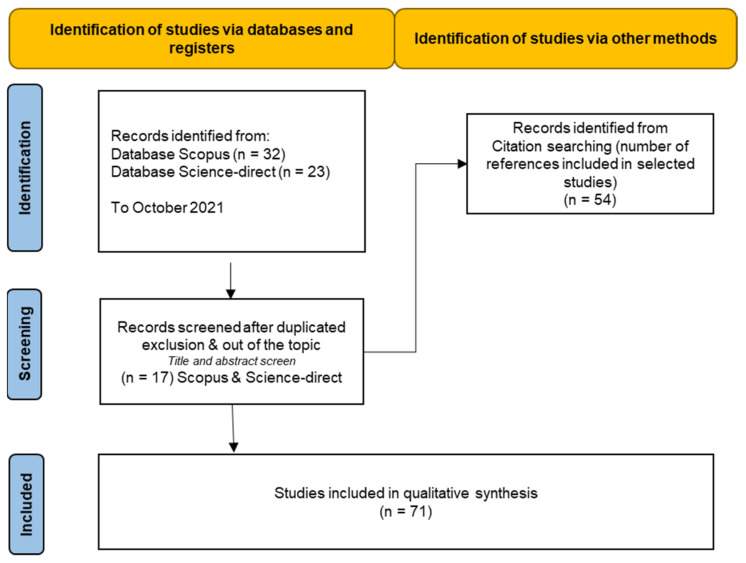
Flow diagram describing the study selection process of the scientific literature.

The review is organized as a research paper. A scoping review was used to synthesize the evidence and assess the scope of the 71 studies on the topic. PRISMA Extension (PRISMA-ScR) approach was used for Scoping Reviews [78]. A comprehensive literature search using Scopus and ScienceDirect was performed in October 2021. Text words and controlled vocabulary for several concepts (Non-thermal, technologies, by-products, fruit, vegetable) within the titles, abstracts, and keywords were used. Only studies published in journals included in Journal Citation Reports (JCR) have been included. Only original research papers (Re) and reviews (Rw) including experimental design and data treatment were selected (Figure 3). This review is structured as follows: (i) the effect of non-thermal treatments on F&V by-products polyphenols; (ii) the effect of non-thermal treatments on F&V by-products pigments; and (iii) the effect of non-thermal treatments on F&V by-products pectin and sulfur compounds.

## 5. Effect of Non-Thermal Technologies on Fruit and Vegetable By-Products Polyphenols 

Table 1 shows the non-thermal technologies applied in F&V by-products focusing on the main findings related to polyphenols. The table is divided in three parts: flavonols, polyphenols and flavonoids. The non-thermal technologies found were: solid-state fermentation, supercritical fluid extraction, ultrasounds, high pressure, hydrostatic pressure, electro-technologies, enzyme-assisted extraction, and light stress. Most of the investigations found (70%) were on fruit by-products or the wine and distillate industry, with only two studies related to vegetables (onion and broccoli) [79,80,81]. 

The recovery of bioactive compounds are mainly affected by varying the solvent concentration (ratio solvent:by-product), applied dose (wavelength, intensity, pressure, frequency), temperature and time (Table 1, Table 2 and Table 3) [82].

**Table 1:** Effect of non-thermal technologies on F&V b-products polyphenols (flavonols, total polyphenols, flavonoids).

	Non-Thermal Technology	By-Product	Findings	Reference
***Flavonols***	Solid-state Fermentation (*A. niger* and *R. oligosporus*)	Plum pomace	Increase of quercentin-3-glucoside (23 to 34 mg/100 g dry matter by *A. niger*; 22 to 24 mg/100 g dry matter by *R. oligosporus*), and quercentin-3-rutinoside (21 to 25 mg/100 g dry matter by *A. niger*) when 2 × 10^7^ spores/g of solid was inoculated and fermentation took place during 14 days at 30 °C	[83]
	Solid-state Fermentation (*A. niger* and *R. oligosporus*)	Plum brandy distilleries waste	Increase of quercentin-3-glucoside (92 to 120 mg/100 g dry matter by *A. niger*; 92 to 110 mg/100 g dry matter by *R. oligosporus*), quercentin-3-rutinoside (42 to 64 mg/100 g dry matter by *A. niger*; 42 to 74 mg/100 g dry matter by *R. oligosporus*) and quercentin-3-galactoside (26 to 36 mg/100 g dry matter by *R. oligosporus*) when 2 × 10^7^ spores/g of solid was inoculated and fermentation took place during 14 days at 30 °C	[83]
	Ultrasound assisted solid liquid extraction	Skins of red and yellow onions	Recovery of quercetin aglycona (118%) after extracted eight times with 20 mL Ethanol (85% *v*/*v*) for 15 min at 25 °C	[34,81]
***Polyphenols***	High hydrostatic pressure	Orange and lemon peels	More intense HPP conditions (500 MPa, 10 min), polyphenols decrease (lemon: 291.08 to 211.95 mg GAE/100 g fresh peel extracts; orange: 400 to 215.31 mg GAE/100 g fresh peel extracts).	[84,85]
	High hydrostatic pressure	Pineapple by-products	Accumulation of bromelain (increase of 350%) and TPC (increase of 36%) at 225 MPa, 8.5 min	[86,87]
	Electro-technologies	Mango peel	Recovery of polyphenols (+400%) at E = 13.3 kV/cm (160 kJ/kg); V = 40 kV (160 kJ/kg)	[54,88]
	Electro-technologies	Olive kernel	Recovery of polyphenols E = 13.3 kV/cm (0–141 kJ/kg), V = 40 kV (0–141 kJ/kg)	[54,89]
	Electro-technologies	Orange peel	Up to 159% in polyphenol extraction recovery after PEF pre-treatment at an electric field densities 1 kV/cm and 7 kV/cm (60 μs, 20 pulses, f = 1 Hz). Recovery of naringin and hesperidin increased ≈2- and 3-fold, respectively.	[84]
	Electro-technologies	Orange peel	Recovery of polyphenols (from 20%, to 159%) for orange peel PEF treated at E = 1–7 kV/cm (0.06–3.77 kJ/kg) + Pressing 5 bars	[54,90]
	Electro-technologies	Papaya peel and seeds	Recovery of polyphenols (>50%) at E = 13.3 kV/cm (160 kJ/kg); V = 40 kV (160 kJ/kg)	[54,91]
	Electro-technologies	Raspseeds stems and leaves	Recovery polyphenols (36–42%) at E = 0.2–5 kV/cm (0–700 kJ/kg)	[54,92]
	Electro-technologies	Raspseeds seeds	Recovery polyphenols (around 50%) at V = 40 kV (0–400 kJ/kg)	[54,93]
	Electro-technologies	Winery wastes and by-products (peel)	Recovery of polyphenols (42%) E = 5–10 kV/cm (1.8–6.7 kJ/kg)	[54,94]
	Electro-technologies	Winery wastes and by-products (pomace)	Recovery of polyphenols (>40%) at E = 13.3 kV/cm (0–564 kJ/kg) V = 40 kV (0–218 kJ/kg)	[54,95]
	Electro-technologies	Winery wastes and by-products (seed)	Recovery of polyphenols (>40%) at E = 8–20 kV/cm (0–53 kJ/kg) V = 40 kV (0–53 kJ/kg)	[54,96]
	Electro-technologies	Fermented grapes pomace	Increase of recovery by 1.2 kV/cm 18 kJ/kg 20 °C (the ratio of total anthocyanins to total flavan-3-ols was increased from 7.1 in non-treated to 9.0 in PEF-treated samples)	[97,98]
	Electro-technologies	Winery wastes and by-products (grapes)	Increase 13% at 0.5 kV/cm, 50 pulses, 0.1 kJ/kg Increase 28% at 2.4 kV/cm, 50 pulses 2.3 kJ/kg	[99,100]
	Electro-technologies	Winery wastes and by-products (grapes)	Increase 34% at 0.7 kV/cm, 200 ms, 31 Wh/kg	[99,100]
	Electro-technologies	Winery wastes and by-products (vine shoots)	Up to 2-fold increase in TPC (Kaempferol, epicatechin, resveratrol) at 13.3 kV/cm, 0–1500 pulses, 50–762 kJ/kg/3 h compared to untreated	[99,101]
	Electro-tecnologies	Citrus peel (orange and pomelo)	Increase of polyphenols recovery, 16 mg/g dry matter for skins (for albedo + flavedo) (E = 10 kV/cm and 50% ethanol solution)	[102]
	Enzymed-assisted extraction	Grape residues	Novoferm^®^ (1:10, 12 h and 40 °C) had the strongest effect on phenolic release (90%) from grape waste (100 mg of dry material was suspended in 1.4 mL of 0.2 M acetates buffer (pH 3.5)).	[60]
	Light stress (UV-B and UV-C; single and combined)	Bimi broccoli leaves and stalks	UV increased initial TPC of leaves/stalks up to 31–97/30–75%, 10 kJ/m^2^ UV-B (UV-B10) + C induced the highest TPC increase (110%) in leaves while UV-B10 and UV-B10 + C led to the highest TPC of stalks after 48 h	[80]
	Optimized supercritical Fluid Extraction	Broccoli by-products	Decrease of polyphenols (<20%) at 400 bars, 40 °C, 5% of ethanol compared with conventional treatment	[79]
	Solid-state Fermentation (*A. niger* and *R. oligosporus*)	Plum brandy distilleries waste	Increase of 3-Caffeoylquinic acid (33 to 53 mg/100 g dry matter by *A. niger*; 33 to 46 mg/100 g dry matter by *R. oligosporus*) when 2 × 10^7^ spores/g of solid was inoculated and fermentation took place during 14 days at 30 °C	[83]
	Solid-state Fermentation (*A. niger* and *R. oligosporus*)	Plum pomace	Increase of 5-Caffeoylquinic acid (22 to 24 mg/100 g dry matter by *A. niger*; 22 to 24 mg/100 g dry matter by *R. oligosporus*) when 2 × 10^7^ spores/g of solid was inoculated and fermentation took place during 14 days at 30 °C	[83]
	Subcritical/critical Fluid Extraction	White grape seeds	Improved recovery of gallic acid, catechin, and epicatechin (>70%) at 1 mL/min CO_2_ flow rate, 20 min extraction, 35 °C, organic modifier density (0.85–0.95 g/mL), modifier (ethanol-methanol: 10–40).	[99,103]
	Ultrasounds	Grape marc	Increase of 11–35% at 24 kHz, 20–75 W/mL	[99,104]
	Ultrasounds	Orange peel (50:50 EtOH:Water)	Recovery of caffeic (207%), p-coumaric (180%), ferulic (192%), sinapic acid (66%), and p-hydroxybenzoic (94%) at 25 KHz, 150 W, 15 min	[84]
	Ultrasounds	Orange peel (20:80; 80:20, EtOH:Water)	Recovery of naringin (38%), hesperidin (42%), TPC (31%) at 25 kHz, 50–150 W, 60 min	[84]
	Ultrasounds	Winery wastes and by-products (grapes)	Increase of 7% (sum of anthocyanins and tannins) at 24 kHz, 5–15 min, 121–363 kJ/kg	[99,104]
***Flavonoids***	Pulsed electric fields	Orange peel	Increase at 5 kV/cm and 20 pulses	[97]

## 6. Effect of Non-Thermal Technologies on Fruit & Vegetable By-Products Pigments

Non-thermal technologies applied in F&V by-products focusing on the main findings related to bioactive pigments are shown in Table 2. The table is divided in four parts: anthocyanins, betalains, carotenoids and chlorophylls. The non-thermal technologies found were: electro-technologies, high pressure, supercritical fluid extraction, ultra [105] sounds, high pressure, and combined techniques (e.g., ultrasounds + enzyme-assisted extraction). It is striking that almost 50% of the research findings are related to vegetables, mainly tomato by-products and others such as eggplant and broccoli. Related to fruits by-products, as expected, the main findings were focused on berries, olive extraction and wineries. It has been recently concluded that berry by-products from processing steps are a cheap and available source for isolating anthocyanins-rich extracts using non-thermal processing technologies as can be observed in Table 2. These technologies have been demonstrated to have unique characteristics such as being effective, rapid, low-cost, and eco-friendly [106,107]. It is essential to highlight that the accuracy of the technique depends on not only the conditions or the matrix of the F&V by-products. For example, the highest recovery of anthocyanin in plum peels was observed after US while in grapes, PEF was the most effective technology [108].

**Table 2:** Effect of non-thermal technologies on F&V by-products pigments (anthocyanins, betalains, carotenoids and chlorophylls).

	Non-Thermal Technology	By-Product	Findings	Reference
***Anthocyanins***	Electro-technologies	Winery wastes and by-products (pomace)	Recovery of polyphenols (>20%) at E = 13.3 kV/cm (0–564 kJ/kg) V = 40 kV (0–218 kJ/kg)	[95,99]
	High pressure	Wine by-products	Recovery of 41% at 600 MPa, 60 min/solvent (50–50% ethanol in water)	[99,109]
	High pressure	Wine by-products	Recovery of 22–83% at 200–600 MPa, 30–90 min, solvent (20–80%; 100–0% ethanol in water)	[99,110]
	Pulsed electric fields	Blueberry pomace (press cake)	Increase of Delphinidin, Cyanidin, Petunidin, Peonidin, and Malvidin. 51%, 71% and 95% at 1 kV/cm, 3 kV/cm, and 5 kV/cm, respectively	[107]
	Pulsed electric fields	Blueberry by-product	Anthocyanin extraction increased (>30%) with PEF process intensification (1–35 kV/cm; 1–10–41 kJ/kg; 10 Hz, 2–100 pulses, 2 μs	[105,106,111,112]
	Pulsed electric fields	Grape by-product (pomace and peel)	Improved anthocyanin extraction (up to 18.9%) at 1.2, 1.8, and 3.0 kV/cm, 18 kJ/kg, 200–2000 pulses, 100 μs	[106,108]
	Pulsed electric fields	Plum by-product	No increase anthocyanins at 37.8–289.8 W, 0.7–25.2 pulses, 10 Hz, 6 μs	[106,108]
	Pulsed electric fields	Peach by-product	Improved anthocyanin extraction (up to 11.8-fold) at 0.8 kV/cm, 0.2 kJ/kg; 0.1 Hz 4 μs	[106,113]
	Pulsed electric fields	Raspberry by-product	Increase 27.5% at 1 kV/cm, 6 kJ/kg, 20 Hz and 20 μs	[106,114]
	Pulsed electric fields	Sour cherry by-product	Improved anthocyanin extraction (up to 54%); 1 kV/cm, 10 kJ/kg, 10 Hz, 20 μs	[106,115]
	Pulsed electric fields	Sweet cherry by-product	Improved anthocyanin extraction (up to 38.4%) at 0.5 kV/cm, 10 kJ/kg, 5 Hz, 20 μs	[106,116]
	Pulsed electric fields	Winery wastes and by-products (grapes)	Increase of anthocyanins: 3-fold at 3 kV/cm 50 pulses; 1.6 and 2-fold ↑ 5 kV/cm 1 ms	[99,117]
	Pulsed electric fields	Winery wastes and by-products (grapes)	Increase of 51–62% at 0.8–5 kV/cm, 1–100 ms, 42–53 kJ/kg	[99,117]
	Pulsed electric fields	Winery wastes and by-products (grapes)	Increased anthocyanin content (1.6–1.9 fold more) at 5 kV/cm, 1 ms, 48 kJ/kg	[99,118]
	Pulsed electric fields	Winery wastes and by-products (pomace)	Increase of Anthocyanins (2-fold more) at 13.3 kV/cm, 0–564 kJ/kg	[99,101]
	Pulsed electric fields + ultrasounds	Blueberry by-products	Increase of anthoycanin extraction (3 fold more) (PEF: 60% ethanol 1:6 and 20 kV/cm; Ultrasounds: 1:6, 40 °C, 60 min at 125 W)	[119]
	Subcritical/critical Fluid Extraction	Grape skin	Recovery of 85% at 100–130 bar, pH of 2–4, 25–30% ethanol, 25–50 mL/min CO_2_ flow, and 3–10% extract flow ratio	[99,120]
	Ultrasounds	Eggplant by-product	US-assisted extraction (15–45 min) was preferable to conventional solid-liquid extraction due to the lower temperature (25 °C) used and higher delphinidin 3-O-rutinoside content (1.5 fold more).	[82]
	Ultrasounds	Jabuticaba by-products	The highest concentration at 1.1 W/cm^2^, 3 min, 10 KHz	[106,121,122]
	Ultrasounds	Pomegranate peel	116 W sonication power with 80% duty cycle for 6 min for extraction of 22.51 mg cyanidin-3-glucosides/100 g pomegranate peel.	[106,123]
***Betalains***	Pulsed electric fields	*Opuntia stricta* peels	Total colorants to ≈80 mg/100 g FW (20 kV, frequency of 0.5 Hz, number of pulses of 50)	[124]
	Pulsed electric fields	Red pricky pear peels	Increase of 2.4 fold colorants (betanin and isobetanin) at 8–23 kV/cm 50–300 pulses + aqueous extraction	[125]
	Ultrasound	*Opuntia stricta* peels	Total colorants to ≈80 mg/100 g FW (400 W power at 24 kHz frequency for 5–15 min)	[124]
***Carotenoids***	Electro-technologies	Olive kernel	Recovery of polyphenols (2-fold more) E = 13.3 kV/cm (0–141 kJ/kg), V = 40 kV (0–141 kJ/kg)	[54,89]
	Microemulsion (Ultrasounds + enzyme)	Tomato pomace	Recovery of lycopene (>20%). The optimal conditions (tomato pomace: double distilled water 1:6): combined ultrasound (20–37 W, amplitude 90% and sonication temperature of 10 °C for 15 min) and enzyme pretreatments (0.2 mL/kg, 30 min, pH 4, 35 °C), saponin as a natural surfactant, and glycerol as a co-surfactant.	[55,126]
	Pulsed Electric Fields	Tomato waste	Recovery of 12–18% of lycopene in acetone and ethyl lactate extracts at 5 kJ/Kg and 5 kV/cm (20 °C).	[55,105,127]
	Supercritical Fluid Extraction	Broccoli by-products	Decrease of beta-carotene (>10%) compared with conventional treatment at 400 bars, 5% of ethanol	[79]
	Supercritical fluid extraction	F&V waste: -sweet potato, tomato, apricot, pumpkin and peach peels -green, yellow and red peppers	Total carotenoid recovery values were greater than 90% *w*/*w*, with β-carotene being the most successfully extracted compound (TCRs 88–100% *w*/*w*), at 350 bar, 15 g/min CO_2_, 15.5% (*v*/*v*) ethanol as co-solvent, 30 min of extraction time)	[128,129]
	Ultrasound	Orange processing waste	Optimization of β-carotene extraction with enzyme assisted technology at 20 kHz, 500 W and 25 °C	[129,130]
	Ultrasound	Red pricky pear peels	Increase of 2.6 fold colorants (betanin and isobetanin) at 400 W 5–15 min + aqueous extraction	[125]
	Ultrasound	Tomato pomace	Lycopene increase (>10%) at 25–40 °C, 0–10 min, 0–100 kPa; 58–94 μm; Hexane %: 25–75	[55,131,132]
	Ultrasounds	Tomato peel	5-fold lower all-trans lycopene content by ultrasounds (30 min 0 °C) compared to thermal extraction (75 °C, 1–2 h).	[14]
***Chlorophylls***	Electro-technologies	Olive kernel	Recovery of polyphenols (>30%) E = 13.3 kV/cm (0–141 kJ/kg), V = 40 kV (0–141 kJ/kg)	[54]
	Supercritical Fluid Extraction	Broccoli by-products	Increase of chlorophylls (>10%) at 400 bars, and 5% of ethanol	[79]

## 7. Effect of Non-Thermal Technologies on Fruit and Vegetable By-Products Pectins and Sulfur Compounds

The main findings about the effect of non-thermal technologies on pectins and sulfur compounds from F&V by-products are detailed in Table 3. The table is divided in two parts: pectin and sulfur compounds. The non-thermal technologies found were: enzyme-assisted extraction, high pressure, electro-technologies, ultrasounds, combined technologies (e.g., ultrasounds + enzyme-assisted extraction) and light stress.

**Table 3:** Effect of non-thermal technologies on F&V by-products pectin and sulfur components content.

	Non-Thermal Technology	By-Product	Findings	Reference
***Pectin***	Enzymes	Apple Pomace	Recovery of 14% by Celluclast 18 h	[133,134]
	Enzymes	Kiwi pomace	Recovery of 4% by celluclast 25 °C 0.5 h	[133,135]
	Enzymes	Passion fruit pomace	Recovery of 2.6–9.2% by Cellyclast 0.5–2 h	[133,136]
	Enzymes	Lime peel	Recovery of 26% by Validase TRL 4 h	[133,137]
	High pressure	Cactus pear peel	Increase of 22% soluble pectin at 600 MPa 10 min	[138,139]
	High pressure	Cactus pear peel	Increase of 9% insoluble pectin at 600 MPa 10 min	[138,139]
	High pressure	Mango peel	Increase of 15% soluble pectin at 600 MPa 10 min	[138,139]
	High pressure	Orange peel	Increase of 59% soluble pectin at 600 MPa 10 min	[138,139]
	High pressure	Passion fruit peel	Recovery of pectin was increased from 7.4 to 14.3% due to HPP pre-treatment. D-GalA of pectin was 65% higher than conventional treatment	[65,140]
	High pressure	Tomato peel	300 MPa pressure performed at 10, 20, 30, and 45 min. 14–15% of pectin recovery at 30 and 45 min	[65,141]
	Moderate electric field	Passion fruit peel	Increase of galacturonic acid (GA) (recovery and content) at 40 min; 100 V; pH 1 (GA); pH 3 (Recovery)	[65,140]
	Ultrasounds	Grapefruit peel	Recovery of 18.2% by 30 °C 10–60 min, 0.2–0.53 W/mL	[131,142]
	Ultrasounds-enzyme assisted extraction without or with hemicellulase or cellulase	Discarded carrots	The pectin was rich in α- and β-carotenes, lutein and α-tocopherol. US-hemicellulase led to the highest pectin recovery (27.1%) at 12.27 W/cm^2^: 20 kHz, 80% amplitude, 20 min	[143]
**Sulfur compounds**	Electro-technologies	Raspseeds seeds	Recovery of isothyocyanates (>15%) at V = 40 kV (0–400 kJ/kg)	[54,93]
	Light stress (UV-B and UV-C; single and combined)	Bimi broccoli leaves and stalks	UV-B (10 kJ/m^2^) + C increased 34% of glucobrassicin levels of leaves	[80]

## 8. Trends and Challenges for Fruit & Vegetable By-Products Application in Food Systems

The incorporation of F&V by-products, their compounds, and/or their extracts/powders can be a relevant strategy for the re-formulation of “Clean Label” ingredients and fortification products. When the non-thermal technologies mentioned above are optimized and applied to F&V by-products, phytochemical bioactive compounds are maintained and/or increased, and then incorporated to food systems. In the following sections, scientific evidence and opportunities for F&V by-product application in food systems are explained: minimally processed fruits, beverages and purees, bakery and confectionary products, food packaging, and cosmetics.

### 8.1. Minimally Processed Fresh Fruit and Vegetables

Minimally processed fresh F&V are commonly defined as any commodity that has been subjected to different processing steps to obtain a fully edible product [144]. Nowadays, the number of emerging technologies using F&V by-products is increasing. For example, a recent study concluded that the fresh-cut apples fortified with lycopene microspheres obtained from tomato peel from the food industry controlled the enzymatic browning after 9 d at 5 °C, enhancing phenolic compounds up to 56% (for chlorogenic acid) after 9 d at 5 °C [14]. In addition, broccoli by-product was incorporated for the enrichment of kale pesto sauce, increasing functional, techno-functional and sensory characteristics [13].

### 8.2. Fruit- & Vegetable-Based Beverages and Purees

A recompilation of the evidences of fortification of beverages by bioactive compounds from F&V by-products was recently published [9]. For instance, the incorporation of the beetroot leaves extract into a veggie smoothie was a potential tool to enrich (50%) phenolic content on the final product. Not only were functional properties enhanced, nutritional and techno-functional properties were also increased [6,89]. Another example is the fortification of coconut water by microparticles of encapsulated grape pomace extract rich in polyphenols [93]. In addition, there is a growing interest in using F&V by-products in fermented beverages for the development of novel functional foods when combining their nutritional and functional characteristics with the enzymatic mechanisms of selected lactic acid bacteria [145]. Enhancing bioactive compounds and other quality parameters could carry out adding F&V by-products to vegetable purée such as tomato puree enriched with grape skin fibers from winemaking by-products [146]. This trend is accompanied by green and non-thermal technologies such as enzyme-assisted extraction, pulsed electric field, ultrasounds, and supercritical fluid extractions [145].

### 8.3. Pasta, Bakery and Confectionary Products

The development of pasta, bakery and confectionary products based on natural ingredients/compounds with antioxidant properties and/or with a reduction of sugars and lipid content is a current trend to obtain new and healthier products. Therefore, the incorporation of F&V by-products is researched such as cookies fortified with purple passion fruit epicarp flour [147], candies fortified with watermelon by-products [148], nutritionally enhanced maize complementary porridges with mango seed and kernel [149], cereal-based foods fortified with by-products from the olive oil industry [150], and spaghetti enriched by persimmon and other vegetal by-products [93,151], among others.

### 8.4. Food Packaging and Edible Coatings

Active packaging presents several options but all of them focus on the addition of additives into the packaging system. The main purpose is to increase food quality and shelf-life. The most common additives are moisture absorbers, gas scavengers, carbon dioxide emitters, antioxidant, and antimicrobial compounds [152]. Related to food packaging obtained from F&V by-products, companies present a special interest due to the interest of circular economy and zero waste strategies around the world during last years. The unique characteristics can be described as follows: (i) to increase antioxidant and antimicrobial activity, (ii) to improve mechanical properties, and, (iii) to protect food products (to increase shelf-life) [153]. Nowadays, F&V by-product components have been proposed to improve the properties of synthetic or bio-based plastic materials [22]. F&V by-products powders and extracts are a good strategy for obtaining packaging with renewable and biodegradable biopolymers, composite films with food stability and barrier properties, active films as carriers of antioxidant and antimicrobial compounds and edible and functional food packaging [22,153].

On the other hand, the colorimetric pH indicator films can be a potential tool for obtaining smart packaging, showing alterations of the food pH by food deterioration and environmental changes. Then, consumers receive authentic information regarding the food’s quality and its edibility (fresh, spoiling, and spoiled product such as milk) [152]. For example, an interesting way to use anthocyanins is building an active use by-date indicator for milk. The development of an anthocyanin-agarose film capable of changing its color in the presence of lactic acid from microbial metabolism has been reported [60].

In addition, a novel technology in which F&V by-products could be revalorized is the use of edible coating, specially containing potent antioxidants and other bioactive compounds from F&V by-products. Up to now, the most common edible coating is chitosan-based edible/biodegradable films because they can extend the shelf life of postharvest fruits. Recent studies have been indicated that coatings enriched with F&V by-products (for example with grape, blueberry and parsley pomace extracts) did not lead to a disruption of the protective function [154]. Natural antioxidants of F&V by-product extracts often contain a high amount of phenolic substances and have been used as active ingredients in the manufacture of active films [152].

### 8.5. Pharmacologic and Cosmetic Uses

The market for natural cosmetics is growing due to the importance of sustainable development and protecting the environment. Manufacturers present an interest in recovering bioactive compounds from F&V by-products for reducing the environmental impact of waste and for converting them into particularly valuable sources of extracts for cosmetic usage [79,155]. In addition, the potential of food and agricultural residues (rich sources of different classes of compounds with valuable active principles) for the preparation of pharmaceutical and bioactive compounds is gaining importance, taking the environmental impact of the overall production process into account [19,156]. For example, the use of broccoli by-products wasted during the preharvest stage were classified as potential ingredients for the cosmetic and pharmaceutical industries, mainly due to the antioxidant effect of its phytochemicals compounds [79]. These findings have been transferred to the industry, and several companies have been recently created, such as https://biodiversocosmetic.com/ (accessed on 25 December 2021).

## 9. Conclusions

Although there is an increase of research focused on the effect of non-thermal treatments on F&V by-products for enhancing phytochemicals and other compounds such as pectin, more scientific evidence is needed to establish the optimum treatments and conditions (extraction, addition, processing, storing, shelf life) for each F&V by-product. Most of the studies were focused on fruit by-products, finding a lack of clear evidence related to vegetable commodities. Even though novel extraction technologies showed a better potential to retain bioactive compounds, the use of improved sustainable methods needs further investigation towards industrial viability (energy consumption, time, equipment, value, cost, etc.). Future investigations should be focused on the effect of ‘green’ technologies in improving the F&V by-products extraction and incorporation for the full utilization of raw materials to preserve a circular economy while enhancing bioactive quality. In this sense, it would be of high interest to optimize nanotechnology for encapsulating extracted bioactive compounds/ingredients, preserving their degradation and optimizing their use efficacy.

## Data Availability

Not applicable.
